# Pain Neuroscience Education Combined with Therapeutic Exercises Provides Added Benefit in the Treatment of Chronic Neck Pain

**DOI:** 10.3390/ijerph18168848

**Published:** 2021-08-22

**Authors:** Norollah Javdaneh, Atle Hole Saeterbakken, Arash Shams, Amir Hossein Barati

**Affiliations:** 1 Department of Biomechanics and Sports Injuries, Kharazmi University of Tehran, Tehran 14911-15719, Iran; njavdaneh68@gmail.com; 2 Department of Sport, Food and Natural Sciences, Faculty of Teacher Education, Culture and Sport, Western Norway University of Applied Sciences, 6851 Sogndal, Norway; 3 Department of Physical Education and Sports Science, Boroujerd Branch, Islamic Azad University, Boroujerd 6915136111, Iran; arash.sh.king@gmail.com; 4 Department of Health and Exercise Rehabilitation, Shahid Beheshti University of Tehran, Tehran 1417935840, Iran; ahbarati20@gmail.com

**Keywords:** therapeutic exercises, pain neuroscience education, chronic neck pain

## Abstract

Background: Chronic neck pain is common in the adult general population. Although the etiology of chronic neck pain is under debate, it is clear that chronic neck pain is multifactorial, with both physical and psychosocial contributors. Objective: To determine whether adding pain neuroscience education (PNE) to therapeutic exercises improved their pain–disability index, pain catastrophizing, fear–avoidance beliefs, and pain self-efficacy in subjects with chronic nonspecific neck pain. Methods: This study was a three-arm randomized control trial. Seventy-two patients with chronic nonspecific neck pain were allocated to three groups: therapeutic exercises alone (n = 24), combined (therapeutic exercises + PNE; (n = 24), and a control group (n = 24). Each program took place three times a week, lasting for six weeks. The disability index, pain catastrophizing, fear–avoidance beliefs, and pain self-efficacy measured by the Neck Pain and Disability Scale (NPAD), Pain Catastrophizing Scale (PCS), Fear–Avoidance Beliefs Questionnaire (FABQ), and Pain Self-Efficacy Questionnaire (PSEQ), respectively. Participants were assessed before and after the six-week intervention, and there was no further follow-up. Results: For the outcomes NPAD, PSC, and FABQ, combined intervention demonstrated more significant improvements than therapeutic exercises alone (*p* ≤ 0.05), whereas no differences were observed between the two intervention groups for PSEQ (*p* = 0.99). In addition, significant differences were favoring experimental groups versus control for all outcomes (*p* ≤ 0.001). Conclusion: Therapeutic exercises combined with pain neuroscience education reduced the pain–disability index, pain catastrophizing, and fear–avoidance beliefs more than therapeutic exercises alone in patients with chronic neck pain. For pain self-efficacy, there was no statistically significant difference between the two intervention groups; however, the combined group had a more significant effect than therapeutic exercises alone. Further studies with longer periods and follow-up are required.

## 1. Introduction

Chronic neck pain (CNP) is a prevalent human problem, especially among office workers [1], with an annual occurrence of nonspecific neck pain that is between 30% and 50% [2,3]. Typically, subjects with CNP have lower neck strength than people without CNP [4], and an association between CNP and reduced endurance and strength in the neck muscles has been observed [5]. More recently, a systematic review indicated that exercises play a significant role in the treatment of CNP, but the relative benefits of any type of exercise should be widely considered [6].

Patients with CNP also tend to have unsuitable pain cognitions, such as fear of movement, pain catastrophizing, and hypervigilance [7,8]. Previous studies have shown that these cognitive factors are related to pain intensity and disability in patients with CNP [7]. The cognitive capacity of patients with chronic pain is reduced compared to a normal population, and changes are dependent on the emotional factors associated with pain rather than the pain itself [9]. For issues related to chronic pain rehabilitation, factors such as pain, beliefs, and attitudes of the patient to pain, fear of pain, fear–avoidance beliefs, and how to manage chronic pain are essential [9]. Studies have stated that fear and avoidance of movement are the best variables to predict chronic musculoskeletal pain over 6 months [10,11]. Pain catastrophizing, fear–avoidance beliefs factor, and movement avoidance due to fear of pain or re-injury are also considered essential factors for prolonged pain and disability [10,11,12]. Therefore, healthcare providers should consider and acknowledge the significant role of psychological factors in working with patients with prolonged disabilities.

However, multimodal biopsychosocial therapy has been recommended for patients with CNP to modify abnormal notions and behaviors, enhance disability levels, and improve the use of self-control skills [13]. The pain neuroscience education increases the patients’ conception of chronic pain and modifies abnormal notions and perceptions [14]. The pain neuroscience education emphasizes explaining the neurophysiology and neurobiology of chronic pain, and pain processing, especially the function of the central nervous system on chronic pain and emphasizing anatomic subjects [15].

Furthermore, there is evidence that a pain neuroscience education can have a positive effect on pain intensity, disability level, fear of movement, and physical efficiency, especially if compound with therapeutic exercises [15,16]. For example, Andias and colleagues [17] examined the effects of pain education and therapeutic exercises in patients with CNP and demonstrated a non-significant reduction in pain. However, the study suffered from a low sample size, which may have resulted in a type II error, and recommended that further research include larger sample sizes. Therefore, further studies are needed to support the clinical application of pain neuroscience education [18,19], or examine if this type of treatment is adequate by itself to modify comprehend disability level [20].

One treatment strategy aimed at helping ease pain, and often the associated suffering and disability, is patient education [21]. During physical therapy care, pain neuroscience education (PNE) aims to help patients understand more about their pain from a biological and physiological perspective. Pain neuroscience education aims to teach patients more about their pain experience from a biological and physiological perspective [22]. Therefore, the present study aimed to compare the effects of adding pain neuroscience education (PNE) to therapeutic exercises on pain–disability index, pain catastrophizing, fear–avoidance beliefs, and pain self-efficacy in patients with chronic nonspecific neck pain. We hypothesized that adding pain neuroscience education to therapeutic exercises would increase treatment efficacy on these variables.

## 2. Materials and Methods

### 2.1. Study Design

This study was a three-arm randomized control trial, with two intervention groups and a control group, and registered at UMIN-CTR Clinical Trial (ID: UMIN000044585). A total of 72 patients were recruited from two rehabilitation and physiotherapy center and by orthopedic physicians through online and offline promotional materials, between March 2019 and April 2020, in Tehran City. Interventions were performed as primary care. The study was conducted by following the Helsinki Convention, and written informed consent was obtained from each patient before being enrolled in this study. Participants were assessed before and after the 6-week intervention. The independent variables were therapeutic exercises, combined intervention (therapeutic exercises + pain neuroscience education), control group, and time (pre-intervention, post-intervention). The dependent variables were pain–disability index, pain catastrophizing, fear–avoidance beliefs, and pain self-efficacy.

Patients with ongoing CNP were recruited from a Rehabilitation and Physiotherapy Center. In the current trial, neck pain was specified as CNP without a specific identifiable etiology, but was provoked by neck postures, neck motion, or palpation of the cervical musculature [23]. Inclusion criteria were as follows: 20–50 years of age, current neck pain, and bilateral CNP for at least three months, with moderate pain intensity (30 to 70 on a Visual Analogue Scale (VAS)). Exclusion criteria were any previous neck or shoulder surgery, fibromyalgia, cervical radiculopathy/myelopathy, history of the whiplash injury, physiotherapeutic treatment in the last three months, and cognitive disorder that prohibited the pain neuroscience education intervention from being followed [24].

**Figure 1:**
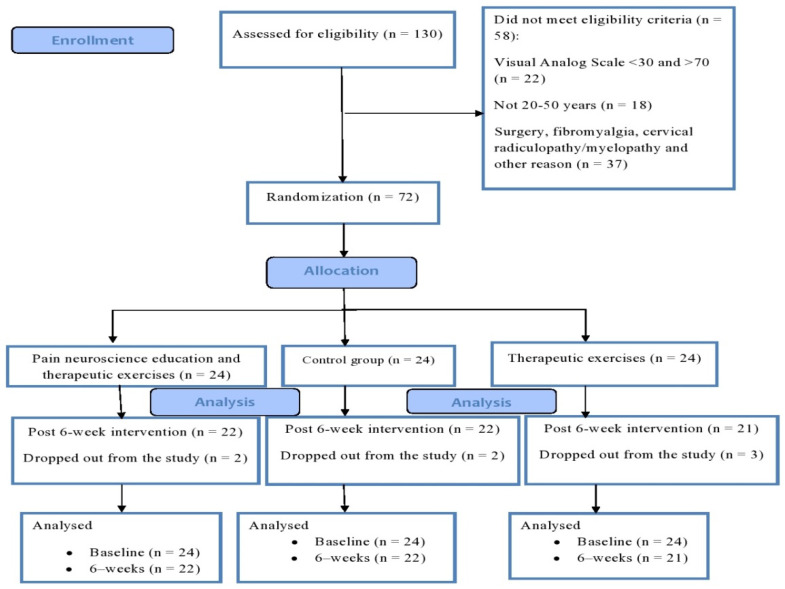
Flow diagram of participants’ recruitment.

A total of 72 patients were registered after sign informed consent. Patients were randomly assigned to the therapeutic-exercises group, combined group (therapeutic exercises + PNE), and control group (Figure 1). For the randomization process, an external evaluator created a random assignment list with a computer program that generated a list of sequential numbers (from 1 to 72). Assignments were placed in a concealed opaque envelope, and opened by the main researcher. A blinded researcher with more than five years of experience in physiotherapy and sports rehabilitation controlled all measurements, training interventions, and inclusion and exclusion criteria. The sample size was calculated by using the G*Power software (v3.1.9.2, Heinrich-Heine-University, Dusseldorf, Germany), using data obtained from a pilot study. The necessary sample size was calculated by using data obtained from a pilot study of 7 subjects (with primary outcome measure: neck pain by VAS). The pilot study showed an effect size of 0.23. Using this data for analysis of variance (ANOVA) with three groups and 2 test sessions, a power of 0.80, and a ɑ of 0.05, a total sample size of 66 was required. An allowance was made for a 10% drop-out rate, increasing the sample size to 72 patients (24 per group).

### 2.2. Outcome Measures

Patients from three groups were assessed for: pain and disability, fear–avoidance beliefs, pain catastrophizing, and pain self-efficacy at baseline and six weeks after. Details of assessment for each of these variables are specified below.

#### 2.2.1. Neck Pain and Disability Scale (NPAD)

The NPAD consists of 20 items. Each item has a VAS of 100 mm with numeric anchors at 0, 1, 2, 3, 4, and 5 (each 20 mm apart). Item scores range from 0 (no pain or limitation in activities) to 5 (as much pain as a possible or maximal limitation). The total NPAD score can vary from 0 to 100 points, and lower values are more favorable [25]. The scale consists of 20 questions relating to 4 domains (neck function, pain intensity, emotion/cognition, and activities of daily living). Studies have reported that the NPDS is a reliable and valid instrument [25,26]. The minimum clinically important difference (MCID) for the NPAD has been estimated to be 11.5 points (0–100) for patients with mechanical neck pain [27,28].

#### 2.2.2. Fear–Avoidance Beliefs

Fear–avoidance beliefs were assessed through the Fear–Avoidance Beliefs Questionnaire (FABQ) developed by Waddell et al. [29]. This questionnaire consists of two subscales. The first subscales include five items that examine pain-induced avoidance views in physical activity, whereas the second subscale includes 11 items to measure the pain-induced avoidance views in regard to work [30]. This scale has 16 items with a 6-point Likert scale (each scored 0 to 6), and the scores range from 0 to 96. A higher score indicates a fear–avoidance belief. The FABQ has been validated and proven to have acceptable reliability and validity for measuring pain related to fear–avoidance beliefs among Persian-speaking patients with acute and chronic neck pain [30].

#### 2.2.3. Pain Catastrophizing

Pain catastrophizing was assessed through the Pain Catastrophizing Scale (PCS). This scale consists of 13 questions (thoughts and feelings). The PCS examines the degrees of 13 feelings or beliefs of experienced painful incidences. [31]. Each item is graded on a 5-point scale (0 = not at all to 4 = all the time). The total score ranges from 0 to 52, with higher scores indicating a more significant pain catastrophizing state. The Persian version of PCS has been proven to have an acceptable level of validity and reliability [32].

#### 2.2.4. Pain Self-Efficacy

Pain self-efficacy was assessed through the Pain Self-Efficacy Questionnaire (PSEQ). This questionnaire consists of 10 items to evaluate the efficacy and adequacy of patients living with pain. The items have a 7-point Likert scale, from 0 (I am not sure) to 6 (I am entirely sure), with maximum and minimum scores of 60 and 0, respectively. Higher scores indicate a strong belief in daily activities while suffering pain [33]. This scale has desirable psychometric features, and it has been reported to have a high level of validity and reliability [33]. Furthermore, the Persian version of the scale has exhibited good reliability and validity [34].

### 2.3. Interventions

#### 2.3.1. Therapeutic Exercises

The compound and development of the therapeutic exercises were designed based on previous studies [35,36] and designed to improve the strength and endurance of the neck and scapula muscles. Each training session lasted 30 to 40 min and included 10 min of warm-up, 15–20 min of therapeutic exercises, and 10 min of cool-down. All training sessions were performed in groups (maximum three patients). The therapeutic exercises were implemented three days per week for six weeks. The progressive exercise training was developed based on sports medicine principles [37]. A detailed description of the exercises is presented in Table 1.

#### 2.3.2. Pain Neuroscience Education

The compound and development of the pain neuroscience education intervention were based on previous studies [17,38,39,40]. Pain neuroscience education discussed peripheral sensitization, central sensitization, and biopsychosocial factors associated with pain [19,41]. During an interview, psychological factors, including self-efficacy, pain interference/disability, coping with pain, catastrophic thoughts, emotional response to pain, anxiety, frustration/anger, fear of damage, concerns regarding pain, and fear of pain, were examined and discussed with patients.

The sessions covered topics concerning the multifactorial nature of chronic pain, sensitization, and plasticity of the brain, aiming at giving patients a better understanding of their chronic pain and thereby engaging the patients in the treatment. [39]. Patients were also taught items such as the physiology of the synapse, the neuron (receptor, axon, and terminal), descending nociceptive inhibition and facilitation (the influence of cognitive factors, notion, motor activities, etc.), central sensitization (receiver field progress, strengthen of the postsynaptic cortex, modify at the cortical and subcortical level, etc.), and peripheral sensitization [40]. A slide presentation (PowerPoint, Microsoft Corp, Redmond, WA, USA) prepared by the instructor was used in all sessions. The first session was a 1-h lecture the first week and then 30–45 min lectures in the following five weeks. In addition, therapeutic exercises were conducted three times per week. Pain neuroscience educations were operated by two physiotherapists who had received the necessary instructions in this field and had more than five years of experience.

#### 2.3.3. Control Group

The control group did not receive any intervention during the study, but was instructed to maintain the proper position at work and home (brochures). After the intervention period, all patients in the control group received a comprehensive rehabilitation program.

### 2.4. Statistical Analysis

The statistical analyses were conducted by using SPSS statistical software (20.0, SPSS Inc., Chicago, IL, USA). The Shapiro–Wilk test was used to check the normality of the information. Descriptive analyses were presented by using mean and standard deviations (SD). Baseline data between groups were compared by using chi-square tests of independence for categorical data, and one-factor ANOVA for continuous data. Repeated Measures ANOVA (RM-ANOVA) tests were used to determine between-subject variables of NPAD, FABQ, PCS, and PSEQ. Moreover, a Bonferroni post hoc test was performed when significant interaction and/or main effect was found. To evaluate clinical significance, 95% confidence intervals (CI) were used. The level of significance was set at a = 0.05.

## 3. Results

One hundred and thirty patients were screened, and 72 were selected and randomized after considering inclusion and exclusion criteria. There was a high degree of adherence to the intervention groups (of the possible 18 sessions, physical-exercise-alone group; 17 sessions (94%), and combined group; 16 sessions (89%). No adverse events were reported. Seven patients withdrew from the study due to personal reasons before completing the interventions (three for the therapeutic exercise group and two for the combined group, and two people for the control group). Table 2 presents the demographic data of all groups. There was no significant difference in demographic characteristics between the groups at the baseline.

The Repeated Measures ANOVA showed a significant effect of time (*p* < 0.001), effect of group (*p* < 0.001), and interaction of time and group (*p* < 0.001) for NPAD, FABQ, PCS, and PSEQ variables. For all measured variables, the effects of both physical exercises alone and combined group (physical exercise + pain neuroscience education) were significantly superior compared to the control group (*p* < 0.05). Furthermore, group with the physical exercises with pain neuroscience education had better scores than the group with physical exercise alone for pain and disability (*p* < 0.001), fear–avoidance beliefs (*p* = 0.041), and pain catastrophizing (*p* = 0.044). For pain self-efficacy, there was no statistically significant difference between the two intervention groups; however, the combined group had a more significant effect on increasing self-efficacy (*p* = 0.99) (Table 3).

## 4. Discussion

This study demonstrated that pain and disability, fear–avoidance beliefs, and pain catastrophizing were reduced, and that pain self-efficacy increased, from using therapeutic exercises alone and combined with pain neuroscience education in patients with CNP. Both intervention groups displayed more significant effects than the control group, and the combined training group (e.g., both pain neuroscience education and therapeutic exercises) was better than therapeutic exercises alone. There was no significant difference in the control group in any of the variables.

The results of this study were both in contrast and supported by previous studies examining the effects of pain neuroscience education [16,17]. For example, Andias et al. [17] investigated the effect of pain neuroscience education and therapeutic exercises in patients with CNP and showed a non-significant reduction of pain. Notably, the average pain intensity at the baseline was lower than the present study and low statistical power, which may explain the contrasting findings. According to the current results, Pires et al. (2015) demonstrated a 51.2% reduction in pain intensity in patients with chronic low back pain after six weeks of pain neuroscience education and aquatic training [16]. Furthermore, the present study showed reduced pain disability in both intervention groups, with the most significant decrement being in the combined group. According to previous studies, a combined treatment consisting of pain neuroscience education and physical exercise may be the best treatment [21,42]. The addition of pain neuroscience education to therapeutic exercises did generate greater effects on pain and disability compared to therapeutic exercises alone. However, the inclusion of pain neuroscience education did seem to be associated with clinical benefit, according to other observed improvements. Previous research has found that pain neuroscience education might be effective for musculoskeletal pain and may reduce disability levels, especially when combined with other manual therapy or exercise strategies [21].

Reduced pain catastrophizing and fear–avoidance beliefs were also more significant in the combined intervention group than the physical exercises alone. The mental modification obtained from pain neuroscience education may generate changes in the quantity and quality of motion [43]. Other studies had observed larger effect sizes reducing the fear of movement when pain neuroscience education and physical exercise were combined [44,45]. Pain neuroscience education also caused a significant reduction in pain catastrophic and fear of movement [46]. Fear of movement is an important outcome, especially when trying to avoid patients going from acute to chronic pain. Significantly, a reduction of fear of movement has been associated with a more significant decline in disability and pain intensity [47]. Pain neuroscience education interventions intend for patients to understand the mechanisms of pain by illustrating that pain is the consequence of sensory hypersensitivity toward a spinal-cord injury [40]. Decreasing the fear of movement may have caused a more practical re-activation, as patients no longer fear worsening their status by doing exercise training. Moreover, reducing the fear of movement may have caused more compliance to physical activity, thus improving pain intensity and disability consequences [48].

Therapeutic pain neuroscience education is an educational approach that intends to explain the synaptic activity, brain processing, and interpretation of chronic pain to patients [49]. Pain neuroscience education is applied to improve the patients’ understanding of the underlying pain physiology and reduce the menace of pain [49]. Specific considerations are intended for the central nervous system and its function in pain-related notions, points of view, and depression, which affect pain perception. One of the essential aims of pain neuroscience education intervention is to alter patients’ beliefs and cognitions regarding their pain experience [50]. Illustrating pain neuroscience to subjects with pain can become turbulent, owed exclusively to patients lacking moderate intellectual ability or those distracted by potent emotion [38]. Thus, this approach attempts to reduce pain and fear avoidance by increasing the patients’ knowledge of why they are in pain.

The present study has some limitations. First, the duration of the present study was six weeks, so a study with a longer course and a follow-up is necessary. Second, it is possible that the main outcomes associated with the cognitive process were not evaluated (i.e., distress and quality of life). Third, the statistical sample size of the present study was small, which may make it difficult to generalize the results to the larger community. Finally, we did not include a group receiving pain neuroscience education alone, which could otherwise have allowed us to investigate the specific effect of pain neuroscience education by comparison with the other groups.

Future research is needed to determine the effects of therapeutic exercises combined with pain neuroscience education in the medium- and long-term. In addition, future studies with large sample sizes should include other variables, such as muscle strength, muscle endurance, cervical range of motion, distress, quality of life, or pressure pain threshold.

## 5. Conclusions

The findings indicate that adding a program of pain neuroscience education to therapeutic exercises led to a greater reduction in the pain–disability index, fear–avoidance beliefs, and pain catastrophizing rather than therapeutic exercises alone in patients with chronic neck pain. For pain self-efficacy, there was no statistically significant difference between the two intervention groups; however, the combined group had a greater effect on increasing self-efficacy.

## Figures and Tables

**Table 1:** Description of the exercises used in the therapeutic exercises group.

Exercise	Dosage	Description
Craniocervical flexion exercise	5–10 reps × 5–10 s	The patients were requested to do a slow and controlled craniocervical flexion task in the supine status. The patient concentrated on feeling the back of the head slide in the cephalad and caudal directions of the supporting surface. The exercises started with five repetitions in each set. Then, in the following sessions, two repeats were added to the repetitions of the previous session.
cervical isometric exercises	5–10 reps × 5–15 s	Isometric neck exercises were performed straight back and forth, to the right and left, with elastic resistance bands. The patients exert force in the opposite direction of the applied resistance. Initially, the exercise started with five repetitions and a maintaining time of five seconds each, and then the number of repetitions and the time gradually increased.
Scapular upward rotation	10–15 reps × 3 sets	The subjects stood with their back against a wall (wall contact from head to buttock) and with the feet shoulder-width apart. In the starting position, the shoulders were abducted 90 °, with the elbows flexed 90 °. The patients were instructed to slide their arms up the wall. The sliding movement ended when the shoulders reached 180 ° of abduction. The subjects were then instructed to maintain the arm position for three seconds. For the first two weeks, they performed only un-resisted exercises. After the first two weeks, exercise was performed with elastic rubber bands. The resistive elastic band was selected from four color-coded resistance levels (yellow, red, green, and blue; The Hygienic Corp, Akron, Ohio), and a gradual overload was applied based on the band’s color.
Backward rocking arm lift	10–15 reps × 3 sets	Initially, the subjects were placed in the quadruped position and instructed to rock backward slowly, until the buttocks touched both heels. The subject was then instructed to lift the arms. For the first two weeks, only un-resisted exercises were performed. After the first two weeks, exercise was performed with dumbbells. Exercises using dumbbells gradually progressed with increasing loads during the intervention period from an initial load of 20% of a 1-repetition maximum and then increased 10% each week.
L to Y	10–15 reps × 3 sets	This exercise was performed on a Swiss ball. The arms were abducted to 90 ° and externally rotated. The elbows were flexed to 90 °, with retracted scapula. The arms were elevated above the head, and the elbows were fully extended so that the arms formed the letter Y. The gradual overload program was performed like the above exercise (backward rocking arm lift).

**Table 2:** Baseline sociodemographic data.

Variables		Groups (No.)			
		Ther Ex (n = 24)	Combined (n = 24)	Control (n = 24)	*p*-Value
Age (year), mean ± SD		31.18 ± 6.37	33.45 ± 7.08	33.70 ± 8.13	0.76
Weight (kg), mean ± SD		80.15 ± 5.10	80.50 ± 4.00	78.23 ± 6.05	0.72
Height (cm), mean ± SD		175 ± 6.15	174 ± 6.50	177 ± 7.68	0.81
BMI (kg/m^2^), mean ± SD		25.05 ± 1.22	25.93 ± 1.45	24.16 ± 1.05	0.79
Duration of pain (year), mean ± SD		3.45 ± 0.84	3.12 ± 0.85	3.76 ± 1.17	0.64
Gender, n (%)	Female	10 (41.66%)	13(54.16%)	12 (50%)	0.47
	Male	14 (58.33%)	11(45.83%)	12(50%)	

Ther Ex = therapeutic exercises, combined = therapeutic exercises + pain neuroscience education, BMI = Body Mass Index.

**Table 3:** NPAD, PCS, FAB, and PSE scores at baseline and follow-up and between-group difference.

Variables	Group	Pre-Training ^a^	Post-Training ^a^	Between-Groups Difference (BONFERRONI Post Hoc Test)					
				Ther Ex vs. Combined		Ther Ex vs. Control		Combined vs. Control	
				Mean Difference (95% CI)	ES (*p*-Value)	Mean difference (95% CI)	ES (*p*-Value)	Mean Difference (95% CI)	ES (*p*-Value)
NPAD (0–100)	Ther Ex	52.55 ± 3.60	35.50 ± 3.80	5.84 (3.09,8.67)	2.30 (0.001 *)	−10.24 (−13.15,−7.34)	3.28 (0.001 *)	−16.09 (−18.98,−13.24)	5.91 (0.001 *)
	Combined	52.86 ± 4.40	23.50 ± 4.83						
	Control	54.63 ± 4.88	53.90 ± 4.09						
PCS (0–52)	Ther Ex	21.50 ± 2.76	15.25 ± 2.55	2.07 (0.06,4.09)	1.85 (0.041 *)	−3.28 (−5.25,−1.22)	2.20 (0.001 *)	−5.31 (−7.38,−3.35)	4.17 (0.001) *
	Combined	21.81 ± 2.90	10.77 ± 2.89						
	Control	22.63 ± 2.61	20.59 ± 3.76						
FAB (0–96)	Ther Ex	48.15 ± 3.80	37.20 ± 4.86	2.92 (0.06,5.78)	2.94 (0.044 *)	−5.89 (−8.75,−3.02)	2.73 (0.001 *)	−8.81 (−11.61,−6.06)	5.83 (0.001 *)
	Combined	50.40 ± 3.45	29.09 ± 3.17						
	Control	49.00 ± 4.15	48.13 ± 3.89						
PSE (0–60)	Ther Ex	25.33 ± 5.84	40.66 ± 5.83	−0.68 (−4.77,3.41)	0.41 (0.99)	8.08 (4.07,12.04)	2.50 (0.001 *)	8.76 (4.80,12.72)	3.52 (0.001 *)
	Combined	23.22 ± 3.84	44.13 ± 5.60						
	Control	24.62 ± 6.25	25.20 ± 5.65						

^a^ Mean ± standard deviation. ES = effect size, Ther Ex = therapeutic exercises, combined = therapeutic exercises + pain neuroscience education, CI = confidence intervals, NPAD = Neck Pain and Disability Scale, FAB = fear–avoidance beliefs, PCS = Pain Catastrophizing Scale, PSE = pain self-efficacy. * Statistically significant difference (*p* < 0.05).

## Data Availability

The datasets analyzed during the current study are available from the corresponding author upon reasonable request.
